# The role of health animators in malaria control: a qualitative study of the health animator (HA) approach within the Majete malaria project (MMP) in Chikwawa District, Malawi

**DOI:** 10.1186/s12913-019-4320-x

**Published:** 2019-07-12

**Authors:** Blessings N. Kaunda-Khangamwa, Henk van den Berg, Robert S. McCann, Alinune Kabaghe, Willem Takken, Kamija Phiri, Michele van Vugt, Lucinda Manda-Taylor

**Affiliations:** 1 The School of Public Health and Family Medicine, University of Malawi, College of Medicine, Blantyre, Malawi; 2 The Malaria Alert Centre, University of Malawi, College of Medicine, Blantyre, Malawi; 3 The University of Witwatersrand, School of Public Health, Johannesburg, South Africa; 4 Wageningen University and Research Centre, Wageningen, The Netherlands; 5 Amsterdam UMC, location Academic Medical Centre, University of Amsterdam, Amsterdam, The Netherlands; 6 Training and Research Unit of Excellence, University of Malawi, College of Medicine, Blantyre, Malawi

**Keywords:** Community health workers, Malaria volunteers, Malaria workshop meetings, Malawi, Social capital theory

## Abstract

**Background:**

Malaria continues to place a high burden on communities due to challenges reaching intervention target levels in Chikwawa District, Malawi. The Hunger Project Malawi is using a health animator approach (HA) to address gaps in malaria control coverage. We explored the influence of community-based volunteers known as health animators (HAs) in malaria control. We assessed the impact of HAs on knowledge, attitudes, and practices towards malaria interventions.

**Methods:**

This paper draws on the qualitative data collected to explore the roles of communities, HAs and formal health workers attending and not attending malaria workshops for malaria control. Purposive sampling was used to select 78 respondents. We conducted 10 separate focus group discussions (FGDs)-(*n* = 6) with community members and (*n* = 4) key informants. Nine in-depth interviews (IDIs) were held with HAs and Health Surveillance Assistants (HSAs) in three focal areas near Majete Wildlife Reserve. Nvivo 11 was used for coding and analysis. We employed the framework analysis and social capital theory to determine how the intervention influenced health and social outcomes.

**Results:**

Using education, feedback sessions and advocacy in malaria workshop had mixed outcomes. There was a high awareness of community participation and comprehensive knowledge of the HA approach as key to malaria control. HAs were identified as playing a complementary role in malaria intervention. Community members’ attitudes towards advocacy for better health services were poor. Attendance in malaria workshops was sporadic towards the final year of the intervention. Respondents mentioned positive attitudes and practices on net usage for prevention and prompt health-seeking behaviours.

**Conclusion:**

The HA approach is a useful strategy for complementing malaria prevention strategies in rural communities and improving practices for health-seeking behaviour. Various factors influence HAs’ motivation, retention, community engagement, and programme sustainability. However, little is known about how these factors interact to influence volunteers’ motivation, community participation and sustainability over time. More research is needed to explore the acceptability of an HA approach and the impact on malaria control in other rural communities in Malawi.

## Background

Malaria remains a significant global problem with an estimated 219 million cases in 2017 and its reduction has stalled [1]. Sub-Saharan Africa accounted for 92 and 93% of the estimated global cases and deaths, respectively [1, 2]. In Malawi, where malaria is endemic, a total of 6.6 million cases occurred in 2017; the parasite prevalence in children under-five years was 24% [1, 3]. Although coverage and use of insecticide-treated bed nets and diagnosis and treatment of malaria has increased for febrile children, behavioural factors may affect prompt care-seeking practices and fidelity in net usage [4, 5]. This diminishes the greater impact of the various Roll Back Malaria (RBM) interventions generally prescribed as part of universal coverage for malaria control [1, 3, 4]. As a result, malaria continues to pose serious health problems and economic burden for communities and health systems in resource-poor settings [6].

Literature and evidence vary on the effectiveness of volunteers or Community Health Workers (CHW) in malaria control programmes in sub-Saharan Africa [7–9]. Studies have reported on the usefulness of using CHWs (receiving a salary) in malaria control programmes (specifically in bed net distribution), diagnostics at community levels and increased immunisation coverage [10–14]. For instance, a study in Uganda described how community-directed treatment with ivermectin for onchocerciasis control with community members improved community participation in decision-making, organisation and resource mobilisation [9, 15]. CHWs have also been mobilised for malarial intermittent preventive treatment for women and children [16], prevention, vector control [17, 18], net distribution [19, 20] control programmes and improving health-seeking behaviour [4, 21]. However, there is little evidence on the role of malaria volunteers without pay in malaria-related interventions.

In Malawi, the Ministry of Health financially supports CHWs as part of a comprehensive community approach to increase access to malaria testing, treatment services and intervention programmes [11, 12, 22]. The largest group of CHWs are Health Surveillance Assistants (HSAs), who hold a secondary school certificate and undergo a 12-week training course [13, 22]. Over 10,000 HSAs are deployed in urban, rural and hard-to-reach areas and provide preventive, curative and promotive health programmes [11, 13]. Malawi has shown the effectiveness of HSAs as the first point of contact under the Integrated Community Case Management for malaria (iCCM) programmes at the community level [10–12, 23]. WHO acknowledges the importance of task shifting, which is defined as a formal or informal redistribution of tasks among health workers to improve the workforce and meet health-related targets [24].

A review of non-financial incentives for all health-workers’ cadres in sub-Saharan Africa argues that there are no studies that focus on how to improve their performance, which is a lost opportunity [25]. A study in Swaziland showed that the most common sources of malaria information were health facilities and not volunteers, despite governmental programmatic support [26]. A study in Zimbabwe revealed that through volunteers, local people had comprehensive knowledge about malaria, but little understanding of transmission, treatment, and prevention [27]. In the Philippines, Barangay Health Workers in rural villages did not contribute optimally to control malaria-prevention activities even in endemic areas due to inadequate training, insufficient logistic support, poorly sustained motivational schemes and a lack of community support [28]. One exception is a study in Uganda by Musinguzi who discusses malaria village health teams (selected by the community members) without a salary who act as a link or connectors between communities and formal health care providers [29].

### The role of health animators in malaria control: the HA approach

There is a call for rigorous studies to evaluate various forms of the redistribution of tasks among formal workforces to inform national programmes, such as malaria control [24]. We looked at the extent to which volunteers recruited from local communities and trained as HAs by The Hunger Project Malawi (THP) became “agents of their development” [30]. It is timely to explore how community members with limited health training and receiving a meagre incentive can contribute to malaria control. It is expected that this approach can be successful if it is integrated into ongoing community development and empowerment. The primary objective of the current study was to understand the influence of using an HA approach in malaria control, and in close collaboration with already existing health care workers.

Many national health programmes acknowledge the essential role volunteers play in the community mainly because they reside in remote and underprivileged communities where communities’ service needs are not adequately met by existing health systems [12]. Volunteers are community-based personnel selected, trained and working in their respective communities [30, 31]. Our focus within this study is on unpaid volunteers (although the term CHW is broad and can refer to both paid and unpaid staff) because there are few published reports about experiences of unpaid volunteers with minimal experience in a resource-poor country such as Malawi. For this study, we adopted Lewin and colleagues’ definition of volunteers as: ‘any health worker carrying out the functions related to health care delivery, trained in some way in the context of the intervention and having no formal professional or paraprofessional certificated or degreed tertiary education’, plus being unpaid [7].

The Majete Malaria Project (MMP) is a multidisciplinary approach to malaria control aligned with the THP programme. Initially, THP worked with HAs in HIV control and therefore were experienced in volunteer work. The concept of the HA approach was re-introduced within MMP and THP as a community-engagement programme on malaria education, learning, implementation, feedback and monitoring activities [4, 21]. The health education approach (education, feedback session, advocacy etc.) used the village malaria workshop meetings as part of the core intervention to reach out to the wider population for malaria control [4, 5].

HAs are volunteers trained in health with an honorary position, who help the community improve health outcomes. For this study, HAs were selected at the village level through a rigorous selection and interview process with local chiefs and members of the community using criteria, including literacy skills, leadership potential and level of motivation. HAs were drawn from the community (ensuring gender balance) and underwent a Training of Trainers (TOT) model of training over one week (two days of general training in skills for community engagement processes and five days of malaria training). This was a ‘tailor-made’ intervention implemented at the community level with the full engagement of malaria volunteers, the local communities and health care facilities. For a comprehensive table on the sessions and topics see [4, 5].

We considered HAs’ communities and social relationships as an asset that could be depended upon for health systems strengthening [32]. We focused on the local organisational structures: 1) leadership, 2) living within the same catchment areas, 3) relatedness of people involved). We held malaria workshop trainings to educate and inform the community about malaria, symptoms, bed-net use and interventions to encourage collective action and promote health behaviour change [5, 33]. We examined HAs’ interactions with other service providers such as HSAs, District Health Management Team (DHMT) including the training outcomes among community members within the HA approach. We explored the role of HAs in malaria control and their understanding of the relevance of the HA approach to draw lessons for rollout in Malawi.

### Theoretical framework: social capital theory

There is increasingly more evidence of the role of social capital, both collective (features of groups) and individuals, in health care utilisation and health promotion [32, 34, 35]. Social capital is defined as the shared values, supports and links that enable individuals and groups to work together [32, 36]. This study reflects on social capital categories including bonding (group networking/shared identities), bridging (action networking) and linking/social networks. These categories provide a useful framework to understand study findings, available support, resources, environments and if and for whom health outcomes are enhanced [34, 37].

**Fig. 1:**
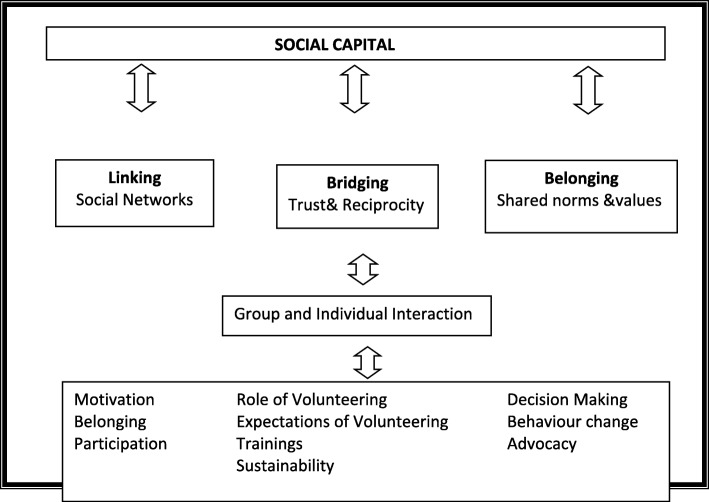
A social capital theoretical framework is informed by Narayan & Cassidy [40]

For **bonding**, we consider and question whether or not people from the same village (with or without support from local chiefs) are encouraged to attend and participate in malaria workshops. For **bridging**, the focus is on the various backgrounds and context-based factors such as age, gender, and education as sources of support and mobilisation at the community level [29, 36]. We also looked at the role of reciprocity in a network that encourages people to expect to receive incentives in community-related and health interventions. The **linking/policy networking** category assesses the communities’ relationship with organisations and formal institutions to ensure coordination and cooperation for the benefit of many [38, 39]. This enabled a critical discussion on power structures, access to resources and inequalities [29, 38]. Focusing on power relationships within formal institutions (THP, MMP, Ministry of Health) and health workers provide a platform to leverage ideas, tensions, resources and relationships for a resource-poor country like Malawi. See Fig. 1 below for the social capital theoretical framework.

## Methods

### Study design

**Fig. 2:**
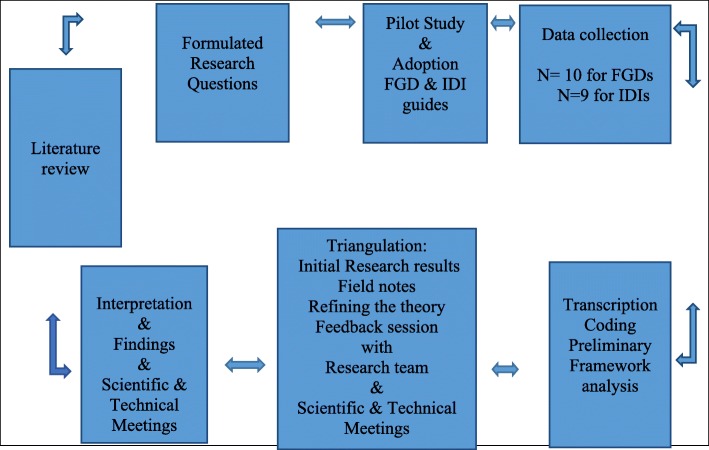
Summary of the qualitative study design

This study used qualitative methods, 10 FGDs with community member and key informants at community and hospital levels to identify nuances within their settings that might influence community members, volunteers and health workers in malaria prevention, diagnosis and prompt care seeking at community levels. The FGDs included group reflections and experiences shedding more light on the roles, experiences of the HAs, HSAs in malaria workshops and local people’s participation and practices in malaria control. Exploratory in-depth interviews (IDIs) with HAs and HSAs provided rich narratives to explain individual and collective actions using HA receiving a meagre allowance in malaria control [41]. Group and individual interviews allowed space for cross-referencing and multiple opinions for triangulation [41, 42]. The study took place between 1 August and 30 November 2017. All selected villages received the HA approach intervention. See Fig. 2, for a summary of the qualitative design.

### Study setting

**Fig. 3:**
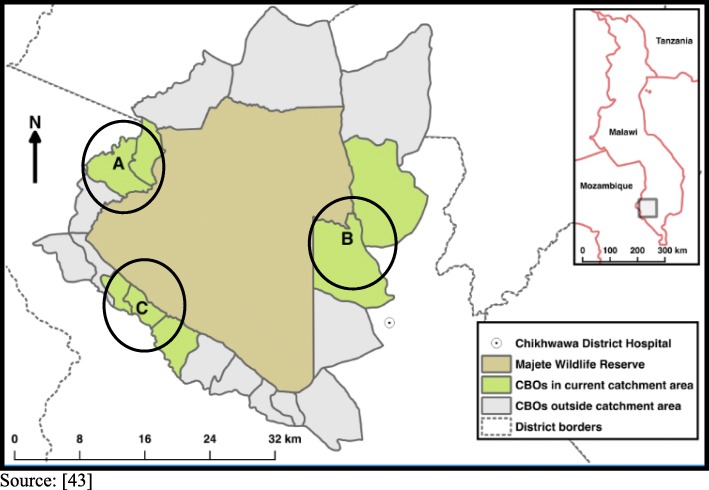
Map of Majete Wildlife Reserve and surrounding communities [43]

The communities surrounding the Majete Wildlife Reserve are subdivided into 19 community-based organisations (CBOs) with a population of approximately 90,000 in Chikwawa district. The study took place in three focal areas (referred to as A, B, and C) covering seven CBOs with a combined population of 24,153 (Fig. 3). The catchment area has been described by Kabaghe et al. but briefly, consists of one district hospital which provides both primary and secondary health care services, and four public and two private health centres providing primary health care [44]. The distance to the closest health facility ranges from 2.5 to 14.7 km for some households [44]. The population is characterised by low educational levels and depends on subsistence and livestock farming for their livelihood [4]. Data were collected in 12 villages (nine villages have only HAs) using a health education approach for malaria control, and three villages coordinated by HSAs under the Chikwawa District Hospitals for triangulation.

### Study population and sampling

The study included a detailed exploration of both individual and group opinion. Nine individual interviews were held with HAs, HSAs and residents who either had or had not attended village malaria workshops in the Majete perimeter. Ten FGDs were held with 6–8 participants per group respondents included district health team members and local leaders (local chiefs, chairpersons of village health committees, representatives from the church and development communities) to triangulate findings at the community level. We used purposive sampling with a maximum variation approach to capture a range of respondents, perspectives and contexts (attendees of village workshops or not, residing within or outside a CBO or epicentre, HAs or HSAs, male or female). This approach provided for a broad range of extremes and reduced gender biases and increased diversity relevant to research objectives [19].

The research team identified the study area using a computer-generated list of names for the intervention areas. The HAs were requested to list households with people who had attended or never attended the village workshops. The names were placed in a hat, and the first ten randomly drawn by the first author were invited to take part in the FGDs. Invitations to attend the FGDs were sent through the HAs and local chiefs for transparency.

### Data collection processes

A semi-structured interview guide led both the individual interviews and FGDs. The guide had nine questions informed by the study objectives and the social capital theory to allow space for probing [41, 42]. Prior to data collection, researchers read the guide questions for familiarisation during the training sessions to identify ambiguous questions that were revised. The tools were further pretested/piloted in non-intervention areas surrounding Chikwawa District Hospital to check for clarity, relevance, comprehensiveness and question flow. Questions identified as irrelevant to answering the primary study objectives were altered or omitted. The first set of questions explored the HAs’ perceptions, roles and experiences with HSAs, community involvement, power relations, change in knowledge, attitudes, malaria prevention practices and treatment including sustainability of the HA approach over time. See Table 1 for an example of an interview guide.

**Table 1:** Interview guide

What is/are the roles of health workers at the community level? Probe on interactions, meetings.	
What is /are the role of HAs in this community? Probe on HSAs, outreach clinics etc	
How do you/communities interact with HAs? Probe on training, village meetings, workshop	
What are some of the successes that you have experienced working with HAs? Probe on failures.	
What types of mosquitoes are found in this community? Probe on Culex or Anopheles.	
How does malaria circulate in this community? Probe on prevention, shared beliefs and treatment.	
Should we maintain the HAs in this community? Probe on ‘why’ and ‘why not.’	
What changes would you like to see when interacting with HAs at community levels?	

The first author and three research assistants collected data in nine villages that used the HA approach and three villages served by HSAs as part of triangulation. All FGDs and IDI’s were held in private spaces. FGDs with community members and opinion leaders were held in a classroom or guardian shelter to explore the collective experiences and views of community members at the community level. The last key informant’s group discussion was held at the hospital for triangulation. IDIs took place in the village clinic shelters in the afternoon to learn about individual volunteer and health worker roles, experiences and challenges. Field notes were continuously recorded, shared and discussed with research team members at the end-of-day meeting briefs as reflections to inform preliminary data analysis. The research team visited two villages per CBO to reduce bias in study findings. Digital recorders collected data and transcribed data were stored as word files in NVIVO 11 in readiness for analysis.

### Data analysis

All qualitative interviews were conducted in English or Chichewa, recorded and transcribed verbatim by the first author and research assistants holding bachelor’s degrees with extensive research experience. The transcripts were shared with the research team and co-authors as part of an iterative process to ensure the quality of scripts and translation. The first author coded the transcripts for both recordkeeping and further analysis in NVIVO 11. Initial analysis began during fieldwork among the first author and research assistants. The first and last author used a framework analysis to analyse the data. Framework analysis is a five-step process that allows analysts to 1) familiarise themselves with data, 2) identify a thematic framework, 3) index (comparisons, identifying quotes representing a specific theme) 4) arrange charts of themes, including 5) mapping and interpreting analysed themes [45, 46]. The first and last author familiarised themselves with the data and shared the coding framework with other research workers for comment. The coding framework included health worker roles, differences, acceptability, and feasibility, challenges, benefits of using an HA approach and sustainability as central themes [47]. The HA approach’s supporting themes included health worker formal and informal tasks, community acceptance, roles and relationships within the community and tensions and fears. The identification of themes and indexing and charting activities were performed in close cooperation of all authors [48, 49].

Result interpretation was iterative with all researchers sending comments at various stages of the write-up [41]. After preliminary data analysis, the results were shared with the implementing partners and collaborators as part of further analysis during a Scientific and Technical Meetings. During all meetings, study progress and results were presented, discussed and further input was provided for in-depth analysis and interpretation of the results. The choice of the quotations depended on the transcripts extracts in NVIVO and if they deepened the understanding of the selected themes and enhanced readability [50, 51].

## Results

A total of 78 respondents participated in 10 separate FGDs: two comprised of health workers, four with community members and the remaining with key informant groups such as opinion and religious leaders. All FGDs included six to eight participants. Nine IDIs included seven with HAs and two with HSAs. See Table 2 for the summary of participants and levels of participation in malaria-related workshops in the study.

**Table 2:** A summary of attributes for the participants

Variable name	Focal Area (n)			
	Focal Area A (23)	Focal Area B (23)	Focal Area C (24)	Hospital (8)
Gender				
Male	9	8	16	4
Female	14	15	8	4
Age				
18 - 24	5	5	3	0
25 – 44	14	14	17	6
>45	3	2	3	2
Education				
None	4	0	2	0
Primary school	7	14	10	0
Secondary School	12	6	12	4
Diploma	0	1	0	1
Bachelor's Degree	0	2	0	2
Master's Degree	0	0	0	1
Level of Participation in workshops				
Never	1	4	2	3
Once	1	1	0	0
Sometimes	5	3	7	5
Often	16	15	15	0
Form of Interview				
FGD	12	12	13	0
IDI	3	3	3	0
KII	8	8	8	8

Nine key themes emerged in our study: perceptions of the HA approach, roles and power interaction with HSAs and members of district health management team, the acceptability of the HA approach at community level, community participation, behaviour change, challenges, sustainability and recommendation for using the approach in poor resource settings. A description of themes is presented in Table 3 below.

**Table 3:** Description of key themes

Theme	Description
Perceptions of HAs and HSAs	Local people’s knowledge and attitudes towards HAs and formal health workers
Roles of HAs and HSAs	Tasks or duties of (in)formal workers including task shifting, relationships, power relations and differences in malaria control
Acceptability of HAs and the HA approach	Community members’ ability to approve, support and work with volunteers
Community involvement	Community perceptions and engagement practices
Change in knowledge, attitudes and practices	Change in malaria practices, transformation and modification of human behaviour due to the HA approach
Challenges	Impediments to the intervention programme, care seeking, facilitation roles, difficult topics during village meetings, strengthening of health systems and advocacy
Sustainability	The prospect of maintaining the HA approach intervention programme over time
Recommendations	Information on how to roll out the HA approach for malaria Control

### Perceptions of the HA approach at the community level

Overall our results show that there is widespread knowledge on the role and work of HAs in the community. Some participants described the HAs’ role as extension workers who helped HSAs share in-depth information on malaria prevention, treatment and linkage to care among villagers. Even people who never attended malaria meetings, e.g., men and old people, knew and supported the HAs’ efforts to encourage pregnant women and children under-five to seek prompt treatment for malaria and use nets at night.

*There are several things that the health animators do in this community since the time they were introduced. Firstly, they have been advocating malaria-related issues to the community members. We also have a committee among the health animators which provides training in malaria prevention methods.* **(4.2 IDI, HA, FAA).**

### Attitudes and experiences of HA in a community-based malaria control project

Almost all HAs had a positive attitude towards their experiences as facilitators for malaria-related workshops at the community level. The HAs’ positive attitude was derived from the social recognition they received as mediators among members of the community, local leaders and village health committees. HAs valued their knowledge gains and career development, which they felt enhanced their communication skills for conveying malaria and health-related topics: symptoms, diagnosis, differences between *Anopheles* and *Culex* (two groups of important mosquito vectors), breeding sites and advocacy for better services. All HAs felt that it was rewarding to receive both positive and negative appraisal during the question and answer, role play and feedback sessions. Furthermore, the provision of a minimum incentive including uniforms, bicycles, bags, and stationery enhanced HAs’ satisfaction with their roles.

*What motivated me is that after we got the training, I felt I should be teaching the people on helping them prevent malaria.* **(3.7 IDI, HA, FAA)**

However, some HAs mentioned personal challenges such as their level of education, which hindered their ability to remember how to facilitate topics on malaria in pregnancy; malaria treatment; responses to questions during feedback sessions; and some clinical tasks. A few HAs yearned for further training to improve their knowledge and scope of practice for recognition by their communities, HSAs and staff at the district level. Refresher training workshops were provided annually for all HAs. Others suggested more refresher-training workshops in the last quarter of every year for reminders about malaria control.

*There are many things I wish I were capable of doing, but the only problem is that I did not go very far with my education. Even though I have the desire to play other roles and activities in the community, there is nothing I can do because of my education status.* **(3.6 IDI, HA, FAC)**

*The health animators need to attend refresher training frequently. Why? Because in a year we go for training once (from January to December), so in between, we forget many things, if they could be taking us twice a year, we could be able to remember things we might have forgotten and tell the people correct information*. **(3.5, IDI, HA, FAC)**

Although one participant bemoaned the lack of frequent refresher training workshops, another participant reported the opposite and said,

*What I am also proud of is if they call them [HA] time and again for training, to remind them, that is the support they need, the other support I see are the bag, books and the bicycle.* **(1.0, Opinion leader FGD, FAB)**

### Complementary and conflicting roles and relationships between HAs and HSAs

Formal health workers such as the DHMT and HSAs appreciated the HAs’ contribution towards malaria control. The idea that HAs were born and raised in the community where they work strengthened their acceptance and relationships with other health workers. Almost all HSAs and community members reiterated that the HA approach ensured the division of labour since there was a value-added workforce to facilitate malaria educational programmes, health campaigns and commitment across communities.

*The health animators are few, but if we compare them with HSA who were employed by the government, they [HAs] are fewer. Having a health animator from the same area, same village, communicating with people is good because he has stayed and grown up with these people. So, it should continue [HA programme].* **(1.0, FGD, Opinion Leader, FAB)**

The formal health workers conceded the training and support provided to HAs as part of the capacity building to strengthen health promotion at grassroots levels. Some HAs and HSAs referred to the combined training on malaria (facilitated by MMP and THP) as strengthening and clarifying their roles and tasks. Joint malaria training workshops by HAs and HSAs was reported as useful because they reinforced the idea of working hand-in-hand to support community outreach programs. Other HSAs valued the cadre and role differentiation, including the HAs’ clear scope of work as running the core intervention and community workshops on malaria.

*People know HA, so they are like role models to the people when they see that one of them, who has grown in their community, is facilitating malaria-related issues. They feel it is essential to take part in the meetings.* **(4.1 IDI, HSA, FAA)**

*Because the health animator stays in our villages, she is the one who helps us minimise problems the area is facing, and if a problem arises that needs to be taken to an HSA, the HA takes this issue to him/her. For example, if there is an outbreak, the HA will tell the HSA, the HSA will organise things and give support to the community.* **(2.4 FGD male & female FAC)**

The HSAs and HAs’ relationship was strengthened as they supplemented skills, knowledge and shared time including activities across communities and during village-level malaria workshops. Some HAs felt that they had limited capacity to handle health-related questions on malaria diagnosis and treatment. In addition, one HSA recommended that supervision for HAs was necessary for motivation and support during question and answer sessions.

*The people in the community feel much better when they see people from different entities of the health sector present at the village workshops*. **(3.6 IDI, HA, FAC)**

*If the members have asked something which is beyond our knowledge, we refer them to the health surveillance assistants for more information. Sometimes the community members ask difficult questions, and this is why we were asked to work with HSAs so that we should always consult them in times when we are unable to answer some of the questions we have been asked.* **(3.6 IDI, HA, FAC)**

Despite the complementary roles of HSAs and HAs, tensions still prevailed in some cases. The tensions were apparent when HAs called for workshop meetings, and HSAs failed to attend. Some HAs mentioned that they felt powerless to invite formal health workers to their meetings. Others reported that they struggled to get attention and recognition from formal health workers due to conflicting expectations and jealousy. HSAs’ failure to attend village-level malaria workshops organised by HAs heightened the latter’s insecurity and affected their facilitative roles at the community level. HAs’ reflections on the possible reasons why formal health workers did not attend the workshop meetings included a lack of motivation, information or invitation for the malaria workshops at a village level.

*Friction is inevitable, like some health workers do not attend workshops because they were not invited for the training, and did not receive allowances. They say if they want us to attend their malaria workshops, they should provide allowances and enjoy the same benefits as HAs*. **(3.2 IDI, HA, FAA)**

Some of the HSAs echoed similar sentiments. They had mixed feelings about allowances, invitations to workshops and material support in relation to the HAs’ level of education, acquisition of skills and growth in status. As a result, some HSAs and community members stopped attending village workshops because they wished that the roles and responsibilities of HAs would rotate every year so that they too could benefit from incentives and social recognition. Moreover, HSAs reported that they also feared the presence of HAs added to their workload and loss of authority in their respective catchment areas.

*This project is a good project, but coordination at the community level is a challenge. We [HSAs] agreed that we should not have HAs leaving their post [to visit households in the catchment area] without my office knowing.* **(4.4 FGD, HSA, CK).**

*So now, HSA’s are taking the program as useless. Whenever we ask them for a gathering or meetings these days, they say we [HSA] are not paid for that.* **(3.1 IDI HA, FAB).**

### Community participation and engagement with HAs during malaria workshops

The village-level malaria workshops were an essential aspect of the project. There were varied responses to the level of participation and interaction in the workshops. HAs had the responsibility to plan, lead and facilitate the topics for the workshops. Respondents had mixed responses on the best or preferred method of learning about malaria and health-related topics. Overall, the results show that the most appreciated sources of learning by most community members included the use of role-playing.

***P10*** *: The best method for the [malaria workshops] is the role play as we take roles.*

***P6:*** *That is true; we can learn through the roles play what causes malaria.* **(2.1 FGD, male & female, FAA).**

***P4:*** *I like how they illustrate all these issues in the stage play. It makes it very easy for me to understand and there some things that we do as a result of lack of knowledge, for example relying on traditional healers when your child is sick. These are some of the fascinating topics.* **(2.3 FGD, male & female, FAB).**

One participant had another suggestion.

***P7:*** *For me maybe if they were doing a class, writing on the board and taking down notes as I may remember later.* **(2.1 FGD, male & female FAA).**

However, community involvement and participation in malaria workshops waned over time because the educational content in each annual cycle was the same. Other community members complained that they had not received any tangible benefits over time. According to the community, just providing the malaria educational approach programmes on malaria symptoms, prevention, care and management was not enough to keep coming to the workshops and as a result, some began to walk away before the end of the workshop.

*So, people are saying ‘since we started in 2014 what is the benefit of this?’ All right we have known about malaria, but you keep on repeating things, there is nothing else you can tell us? Should we just be hearing only about malaria and sleeping under a mosquito net?* **(3.3 IDI, HA, FAB).**

*Most of the time, it happens that maybe I have started the facilitation, some people start walking away before the meeting is closed, after hearing the [repeated] topics I am covering they just heading back home; this disappoints me but not much, I continue with the few.* **(3.4 IDI, HA, FAB).**

As for other factors contributing to non-attendance, some participants pointed out that men are breadwinners who spend their time on farms or seeking piecework. Hence, they do not have the time to spare for workshops. HAs referred to the absence of local leaders; village chiefs’ delays calling for fortnightly meetings; miscommunication between chiefs and communities; and rain. Women mentioned sickness, funerals, not receiving nets and working on farms as their primary reasons for not attending village-level malaria workshops. In essence, another significant challenge that impacted community involvement in malaria-related activities was also due to the study’s randomisation for participants which affected further their attendance at the malaria meetings.

*Some of the community activities affect the work of the HA. For example, if there is a funeral in the village, the HA may not be able to do their work or conduct the village meetings. Weather changes are also one of the major issues that impede the work of HA in the community. In cases where the community is receiving too much rainfall, HA is not fully operational because some of the rivers are flooded. I think these are the significant challenges that impede the HA’s work in the community.* **(4.2, IDI, HSA, FAA).**

*We have not had any problems with health animators. However, we have realised that some of the people in other communities have been provided with window screens and helping them manage the swamps surrounding their homes to ensure full prevention of malaria. This kind of intervention has not been happening in our communities [receive malaria education only], and this makes me sad*. **(2.3 FGD, male & female, FAC).**

### Attitudes, behaviours towards malaria prevention and health-seeking practices

Community involvement was a vital factor that supported the duties of the HAs and encouraged a change in knowledge, attitude and behaviour for malaria prevention and treatment. Participants reported that they were able to link up with their local leaders and village health committees to ensure that areas surrounding households were maintained. Other participants reported that some communities moved from using cow dung to repel mosquitoes during the night to using mosquito nets. Some women reported that they stopped visiting the traditional healers as a way to comply with malaria early diagnosis and treatment.

*There are now improved cases of treatment seeking as in comparison to the past when before the health animator intervention. People are aware of the importance of seeking treatment at the first signs and symptoms of any illness.* **(3.6 IDI, HA, FAC).**

*The HA tells us that we should cover the pools of water because mosquitoes live there. They taught us how to assess and know when the child is suffering from malaria for example fever; if he/she is vomiting, it means he/she has malaria we should rush to the hospital.* (**2.4 FGD male & female, FAC).**

*Before we got this message, in the beginning, we were using dung. We were burning the dung around the place we want to sleep so that mosquitoes should fly away. We did not have nets, these are new, we were burning dung, and the smoke would be circulating in the house and mosquitoes were flying away. Another thing is that, according to our culture, before the HA[s], when a child was sick, with fever, we did not know that it is malaria. We dug a tree. Our ancestors showed us that when you see a child vomiting, this is the medicine, dip this in water and give the child, we were losing a child.* **(I.3, Opinion leaders, FAC).**

### The role of HAs in advocating for better health services

Some community representatives argued that they present their stock-outs or poor service provision as challenges to the HAs. The HAs become the link to other health workers and local chiefs, thus promoting self-reliance at the community level. However, most community members reported that they handled drug stock-outs by doing nothing, buying from a shop or seeking medication from private clinics.

*If maybe our [health] animators were to have a committee that’s responsible for collecting complaints it would be good as we would report our complaints and they would be getting to you or whoever is responsible for the animators? However, on our own, ah, [we cannot present our complaints] but [it is possible]at the village level, even at Group Village Headman level.* **(2.6 FGD male & female FAC).**

*There is nowhere we take our complaints. If there are no drugs in the hospitals, all we are asked to do is go to private clinics where we can buy drugs. However, we have Area Development Committee (ADC) members in the community, and sometimes these members take our complaints to the senior chief* [name] *who is a bridge between the community and the government officials. However, apart from that, there is nothing the community can do about the complaints*. **(2.3 FGD, male and female FGD F. Area B.)**

### Sustainability

Almost all community members, opinion leaders and formal health workers strongly accepted the HA approach and continue to support it because it was part of the routine monthly programmes they became accustomed to attending. Members of the community were confident that the training and skills that HAs had received would continue being shared over time and sustain the programme. The HAs echoed the same sentiments of receiving knowledge and skills as part of capacity building efforts to enhance malaria prevention and control over time. Both the HSAs and HAs mentioned that they could sustain the programme by working together during clinic activities.

*Those of us who are learning are supposed to keep these things in our heads as our treasure because knowledge is treasure … project people are going, but we know our heads; we will continue to teach*. (**1.0 FGD, opinion leader, FAA).**

*P4: We support them by making ourselves available at the workshops.*

*P5: There is nothing we have done so far to support their work, maybe we will start thinking about building small clinics for the health animators so that they can have a proper base of operations in the future*. **(1.3 FGD, opinion leader, FAC).**

The opinion leaders also mentioned that they provide monthly support by using the chief’s aides to call for meetings at the *bwalo* [central area] on behalf of HAs. Some chiefs provided support to HAs in other village meetings to share malaria messages. The active involvement of local chiefs was discussed as key to the success and sustainability of the programme.

*We advised them to use the under-five clinics; HAs are welcome to address the caregivers. There, 15 min will be enough not only that you can visit schools, church gatherings even village meetings, but they can also come and deliver the message.* **(4.3 FGD HSA, FAB).**

*For example, we do have a friend from NGOs. Whenever they want to deliver a message they do come and ask for clinics dates, then book a day to come and give out the message whether it is from village x, village s or bwalo they do visit us. So, they can copy that and do the same in 10 to 15 min. They can be given. It will be enough for them since we already have time for a health talk with the community members.* **(4.3 FGD HSA, FAB).**

However, other health workers at the district level and opinion leaders argued that the linkage between the HSAs and other formal health workers is not strong enough to ensure sustainability. They mentioned the need to continue playing complementary roles by holding health education and feedback sessions together. Male participation was reported to be low in some meetings because of the educational approach and lack of incentives. Some members of the communities requested incentives such as developmental programmes or distribution of food rations be integrated with malaria programmes to ensure sustainability.

*If there is a possibility to strengthen the relationship between the HAs and HSAs [it] is so they can work hand in hand, where one is falling short another can help out, so they can work towards a common goal.* **(1.2, FGD, opinion leader, FAB).**

*If you were to use soya [as an incentive], you would see how many people attending the malaria workshop; you would not have been able to send them back.* **(2.1 FGD, male & female, FAA).**

## Discussion

This study contributes to the body of literature on the roles of volunteers in malaria control [7, 9, 29, 30, 39, 52]. We documented and analysed the influence of the HA approach enhancing community capacity and action with improved health behaviours and strengthening of malaria intervention programmes. There is high awareness of and positive attitudes towards working with malaria volunteers, known as HAs, to complement malaria intervention programmes. However, the HAs’ minimal education level affected community attendance at malaria workshops, which affected their motivation [53]. We reflected on the social capital framework, specifically bonding, bridging and linking forms to extrapolate how group cohesion, a shared purpose and power relations influenced malaria workshop attendance, health worker roles and community involvement and outcomes [54, 55]. The HA approach, as a community and peer-education-based approach, ensured community understanding and laid a platform to encourage behaviour change.

### Bonding and bridging with malaria control

This study revealed that the HA approach bonded community health volunteers and their communities through a common problem of malaria and their training and knowledge on the modes of malaria transmission and prevention. The communities gained not only technical skills but also social skills and actions (talking in public, debating, role play, planning, communicating with leaders), which increased confidence, changed the prevailing mindset and improved collective action. The HAs served as a ‘bridge’ when they encouraged individuals and local chiefs to participate in village-level malaria workshops and role-plays, which provided opportunities for malaria awareness including better prevention and early diagnosis and treatment seeking. The HA approach as a community and peer-education based strategy ensured community understanding and laid a platform to encourage behaviour change related to using bed nets and or accessing traditional healers. As noted in a systematic review in Sierra Leone, health education outreach visits and reminders are useful in for ensuring collective action and strengthening malaria prevention intervention programmes [56, 57].

Our study shows that there is a readily available human resource at the community and district level to take up the roles of volunteers or supervisors, respectively. McMahon discusses the dual role of volunteers that enables community action while harbouring their concerns and fears [57]. Similarly, the HAs’ relationships with district health staff and HSAs in this study provided the basis for knowledge sharing, training and monitoring framework. The HAs acknowledged playing complementary roles with formal health workers (HSAs) in their respective catchment areas as part of strengthening their relationship and sources of support in training, meetings and health campaigns. However, in some cases, the tension was recorded between HAs and HSAs. For instance, the HSAs failed to attend village workshops after being invited. In addition, the differences in the cadre between the HAs and HSA were both a facilitating factor and barrier to a sustainable working relationship and community health outcomes. This mirrors findings from two studies by Mcmahon and Angwenyi which mention a superiority complex of formal health workers, tensions with volunteers and community engagement in research interventions [53, 58]. Having CHWs with minimal education level and no certificate can lead to conflict over remuneration, competency, and support from communities and health facilities staff [53, 57]. There is a need to continuously hold refresher courses for both volunteer HAs and paid CHW, such as HSAs, to ensure transparency and a sustainable working relationship over-time.

Bridging social capital to attain a shared objective or a set of shared purposes was one of the broader aims of introducing the HA approach. The MMP brought volunteer groups (HAs) and communities together with medical groups (formal health workers) and community leaders who were willing to send their officials to help HAs call for workshop meetings. In other areas, the leaders presided over the meetings with HAs and health workers to encourage a collaborative effort in intervention programmes [59]. Action networking of this kind added educational support and skills for the implementation of interventions at the community level, which could benefit national and global-level initiatives to control malaria [55]. In our study, communities informed us that the HAs and the local chiefs were perceived as their advocates on health-related issues. Hence, most community members reported that they depended on the HAs to be their voice when advocating for better health service provision.

HAs’ malaria workshops in various communities were one way of encouraging participation and creating community action plans and problem analysis sessions to help reduce the burden of malaria in the community. By implementing the HA approach, it was hoped that communities would isolate challenges, increase demand for health services and promote positive behaviour change (advocacy) including sustainable ways to address challenges. However, the communities demand better health services was limited, and participants pointed out that when the health centres experienced drug stock-outs, people would simply resort to other means to obtain medicines. This type of disengagement with community members and local health officials only served to undermine the growth of collective advocacy efforts [6, 59]. McMahon and colleagues mention a comprehensive package for employing leaders, local people, and health workers to be involved in administrative tasks, manual labour and mediating between communities and the health system, which may be an option in Malawi [57].

Linking social capital builds on the social cohesion created through action taken in networks to foster change [54, 55]. Our findings demonstrate that linking social capital was exemplified by the interaction and relationships among THP, MMP, local chiefs, the district and the national health providers. The HAs were recruited at the community level with the help of local chiefs and THP support. The training and facilitation of technical malaria information, education and communication materials came from other partners, the Health Education Unit and MMP. The MMP and THP provided the funding for HAs’ training (including refresher training) and support (i.e. monitoring meetings, stationery, bicycles, manuals, t-shirts, and bags). This was in-line with recommendations from Strachan and colleagues [30, 60] who emphasise the need for assessing enabling conditions within a community to sustain intervention programmes. Moreover, Strachan et al. and WHO described the benefits of learning and adapting programmes informed by local progress data and meetings since one size does not fit all [1, 60].

### Sustainability of the HA approach: community involvement and incentives

The village workshops allowed HAs to have a platform to exercise their facilitation skills and for communities as beneficiaries to plan, debate or role-play, ask questions and enhance their knowledge on malaria prevention and treatment. However, boredom due to the repetition of malarial sessions into the final year proved to be a barrier to continued community workshop attendance. Others began to walk away from meetings the moment they heard a topic repeated, to the dismay of HAs. Others stopped attending completely. The bonding and shared purpose that initially inspired communities’ collective support and commitment in village-level malaria workshops began to wane over time. In addition, communities complained by asking why randomisation of the malaria intervention trial was not coming to an end so that they too could benefit from additional interventions such as house improvements and larval source management [5]. Kane et al. suggest that there is a need to continuously provide new knowledge for the sustainability of a project despite the cost implications [61]. Similarly, the provision of comprehensive programmes considering resources, context, competencies and support systems ensures the sustainability of community-based programmes [9, 62].

A study by Glenton et al. argues that the provision of monetary and non-monetary support in intervention programmes through collaborating partners can result in mixed outcomes [8]. Our findings also show that the material and monetary incentives HAs received during their training had the potential to undermine the sustainability of the HA approach. The HAs in our study were enthusiastic about the training sessions, which brought allowances, training manuals, bags and bicycles for transportation. The influence of positive feelings and motivation at individual and contextual levels are consistent with studies done in Malawi [4, 10, 11], Tanzania [17] and Zambia [63] where interventions with short-term and focused training only led to better implementation processes for a while since they were donor-dependent.

However, financial and material incentives can also be de-motivators for volunteers [53, 64]. This is evidenced by comments made by some opinion leaders, formal health workers and some members of the community who were envious showing that the HAs’ position was a sought-out role since it provided not only an acquisition of skills, personal growth, social recognition, support, but also allowances. The dual role of HAs as community members and a ‘bridge’ between communities and health workers could also be compromised in some cases as tensions resulted in the lack of support that impacted on the volunteers’ work and affected sustainability. Building long-term, trusting relationships and strengthening community and volunteer empowerment (knowledge, competence, values, impact), can improve the desired health outcomes [57, 61].

Other studies mention the mixed outcomes of linkages or networking with partners and the perceived benefits (material and immaterial that volunteers receive) [30, 65]. Some training workshops and refresher courses have been a source of income-generating programmes that ensured retention of volunteers and community health workers in Mwanza, Malawi [13]. Others show how short-term training under iCCM has led to a reduction in community confidence in lay health workers [16]. In our study, bridging and linking processes facilitated financial support, training, new ideas for advocacy, critical thinking and problem-solving among participants attending malaria workshops. Unfortunately, for some caregivers, the fear of reprisal when accessing services stopped them from requesting for better service provision and care. Hence, we agree with various studies in Malawi and community-based intervention programmes that highlight the need to recognise incentives and disincentives based on a contextual level for sustainability [13, 23].

### Limitations

This study shows that outcomes of the community approach for malaria intervention are determined by context and inter/intra-relationships that vary over space and time. Our evaluation of the role of the HAs and community participation included people living in only the intervention areas. Hence, there may be bias in our interpretation of the results since we did not use control areas for comparison. In addition to the sampling frame bias, the evaluation took place in the final year of the intervention period. Hence, there may be issues related to social and recall bias due to elapsed time. This study adopted the social capital theoretical framework including bonding, bridging and linking perspectives. Tying our analysis to the three perspectives above to explain the process and causal factors may be reductionist; however, this strategy provided a platform to inform our evaluation on the role of HAs in malaria control [66].

## Conclusions

Based on the study findings, it is apparent that malaria-related community-led interventions require deliberate and constant community engagement activities for the “return on investment” (ROI) in training HA, organising workshops so that they can support health care workers. The HA approach is a useful strategy to complement malaria prevention strategies in resource-poor settings in Malawi. The HAs are valuable volunteers who offer complementary functions and efforts for malaria management and control at the community level in endemic malaria countries. Our findings show that through the bonding, bridging and linking networks established through the HA approach, collaboration with traditional authorities, and informal health workers buttressed community involvement, interactions, and engagement practices. Technical and social skills to present topics in public and role-playing malaria-related topics enhanced communities’ knowledge and practices. More caregivers sought prompt and appropriate malaria care, thereby stopping their visits to traditional healers. However, in those cases where HAs lacked support from formal health workers and community members in their involvement and engagement during malaria workshop activities, we saw a decrease in the functions they were to perform. The HA approach relies on continued support for training and quality assurance.

Despite the limitations in this study, the HAs in Malawi are well positioned to provide support for malaria control. Key stakeholders in malaria control need to consider factors such as the roles of malaria volunteers, educational levels, community expectations, incentives, and supervision as key issues in the implementation of the HA approach. There is a need for comprehensive malaria health education packages including prevention and control modules to sustain the role of the HAs and community participation over time. A multi-sectoral approach from partners and traditional authorities is key to sustainable malaria programming. There is potential to scale up the HA approach; future studies should consider integrating it into a child, maternal or mobile clinics to provide malaria prevention and control across villages in malaria-endemic areas in the country.

## Data Availability

Data from this study will be made available upon request from Blessings Kaunda-Khangamwa at b.n.kaunda@gmail.com. If one needs to use the data, they will need to seek approval from the Majete Malaria Consortium and their review boards.

## Abbreviations

CBO: Community based organisation
CHW: Community Health Workers
DHMT: District Health Management Team
FGD: Focus group discussion
HA: Health Animator Approach
HAs: Health animators
HSA: Health Surveillance Assistant
IDI: In-depth Interviews
MMP: Majete Malaria Project
THP: The Hunger Project
